# *smarce1* mutants have a defective endocardium and an increased expression of cardiac transcription factors in zebrafish

**DOI:** 10.1038/s41598-018-33746-8

**Published:** 2018-10-18

**Authors:** Jorge Castillo-Robles, Laura Ramírez, Herman P. Spaink, Hilda Lomelí

**Affiliations:** 1 Departamento de Genética del Desarrollo y Fisiología Molecular. Instituto de Biotecnología, Universidad Nacional Autónoma de México, Avenida Universidad 2001, Cuernavaca, 62210 Morelos Mexico; 2 Institute of Biology, Leiden University, Leiden, 2333 BE The Netherlands

## Abstract

SWI/SNF or BAF chromatin-remodeling complexes are polymorphic assemblies of homologous subunit families that remodel nucleosomes and facilitate tissue-specific gene regulation during development. BAF57/SMARCE1 is a BAF complex subunit encoded in animals by a single gene and is a component of all mammalian BAF complexes. *In vivo*, the loss of SMARCE1 would lead to the formation of deficient combinations of the complex which might present limited remodeling activities. To address the specific contribution of SMARCE1 to the function of the BAF complex, we generated CRISPR/Cas9 mutations of *smarce1* in zebrafish. Smarce1 mutants showed visible defects at 72 hpf, including smaller eyes, abnormal body curvature and heart abnormalities. Gene expression analysis revealed that the mutant embryos displayed defects in endocardial development since early stages, which led to the formation of a misshapen heart tube. The severe morphological and functional cardiac problems observed at 4 dpf were correlated with the substantially increased expression of different cardiac transcription factors. Additionally, we showed that Smarce1 binds to cis-regulatory regions of the *gata5* gene and is necessary for the recruitment of the BAF complex to these regions.

## Introduction

The SWItch/Sucrose Non-Fermentable (SWI/SNF) complexes are ATP-dependent chromatin remodeling machines that are evolutionarily conserved from yeast to humans. Their enzymatic function involves remodeling nucleosomal DNA, thereby facilitating the binding of transcriptional factors to nucleosomal templates^1^.

In vertebrates, at least 28 genes encode subunits of the SWI/SNF complex. SWI/SNF complexes (often referred to as BAF) can form various assemblies from homologous subunits. The subunit composition and stoichiometry of BAF in different cells change dynamically during differentiation and distinct combinations have been associated with specific developmental processes^1–5^

The central catalytic activity of the BAF complex is ATP hydrolysis. This activity is achieved by the Brahma (BRM/SMARCA2) or Brahma-Related Gene 1 (BRG1/SMARCA4) subunits. Three other subunits (BAF155/SMARCC1, BAF170/SMARCC2, BAF47/SMARCB1) together with BRG1 or BRM are sufficient to reconstitute a core complex capable of remodeling mononucleosomes *in vitro*^6^. Additionally, other subunits help to target the complex to specific genetic loci templates^1,7,8^. Mammalian BAF complexes are further subdivided into BAF and PBAF (Polybromo-associated BAF) complexes: BAF complexes are defined by the AT Rich Interactive Domain 1 (ARID) containing subunits ARID1A or ARID1B, while PBAF complexes include BAF200 as the ARID-containing subunit. Besides, PBAF only utilizes SMARCA4 as the ATPase and incorporates BAF180/PBRM (polybromo), BRD7 (Bromodomain containing 7) and BAF45a^9^.

In recent years subunits not found in yeast have been identified. SMARCE1 is an animal-specific subunit, which is present in all BAF and PBAF assemblies. Its main structural feature is a high-mobility-group (HMG) domain, which promotes binding to four-way junction DNA^10^. Extensive work has demonstrated a specific role of SMARCE1 in the recruitment of BAF complexes to endogenous nuclear receptor targets.^11,12^. SMARCE1 also interacts with a wide range of protein partners outside the BAF complex. For example, in the mouse embryo, SMARCE1 is responsible for the silencing of the CD4 gene during T cell differentiation^13^, and during the repression of neuronal genes in non-neuronal cells, SMARCE1 interacts with the transcriptional co-repressor Co-REST and facilitates repression^14^. In humans, it has been shown that mutations in *SMARCE1* predispose to meningioma disease^15,16^ and to the Coffin-Siris syndrome^17^, and thus, this gene has been connected to malignancy.

The BAF complex has been widely studied in the context of mammalian heart development. In mice, mutations in different subunits lead to defects in heart development and cause embryonic death. Among the mutated genes that produced cardiac anomalies *in vivo* were SMARCA4^18^, BAF60c/SMARCD3^19–21^, PBRM^22,23^. SMARCA4 function has also been examined in zebrafish embryos. Studies in both organisms revealed that the relative levels of cardiac transcription factors and BAF complexes are important for proper cardiogenic expression^18^. On the other hand, SMARCD3 is a cardiac-enriched subunit of the BAF complex that assembles in a mutually exclusive manner into BAF to become part of what has been considered a cardio-specific cBAF complex^24^. The cBAF complex promotes cardiomyogenic differentiation activity in mouse and zebrafish^25^. Previous work attempting to assess the relative importance of BAF and PBAF complexes in heart development, indicated that both complexes are functional during this process, suggesting that different assemblies of the SWI/SNF complex with distinct spatial or temporal remodeling activities may co-exist in the developing heart.^22,26^.

This study focuses on Smarce1 function in zebrafish development. SMARCE1 is a constitutive component of all BAF animal complexes, but it is not a core subunit. It is known that complexes with mutations in the HMG domain of SMARCE1 can still bind DNA and mediate ATP-dependent nucleosome disruption^10^, however the SMARCE1 HMG domain likely provides additional specificity during target recognition by the BAF complex. Therefore, the loss of this subunit would be expected to disable important functions of the BAF complex. SMARCE1 inactivation is not redundant with SMARCA4 loss of function, since in SMARCE1 mutants the ATPase activity of the BAF complex would be intact. The understanding of the specific functions of SMARCE1 is important because it has been noted that it exhibits relevant interactions with complexes involved in gene repression^13,14,27,28^, which leads to the question of whether the loss of SMARCE1 has different effects on particular properties of the BAF complex, for example repression versus activation. Zebrafish are a good vertebrate model to ask these questions because due to maternal inheritance, fish might survive longer with a decreased chromatin remodeling activity. To analyze the function of Smarce1 we generated CRISPR/Cas9-mediated mutations in the zebrafish gene. We found that the loss of Smarce1 produced a strong phenotype. At 72 hours of development, *smarce1* mutants presented smaller eyes, body curvature defects and pericardial edema. We showed that mutant embryos have heart defects including dysmorphic chambers, absence of looping, slower beating and absence of circulation. These defects were accompanied with an abnormal morphology of the endocardium, which was detected from 20-somite stage and an augmented cardiac gene expression detected from 48 hpf. ChIP analysis demonstrated that Smarce1 directly binds to *cis*-regulatory regions of *gata5*, a gene that is highly upregulated in the mutant.

## Results

### CRISPR/Cas9 mutant alleles of *smarce1* have strong morphological defects at 72 hpf

**Figure 1:**
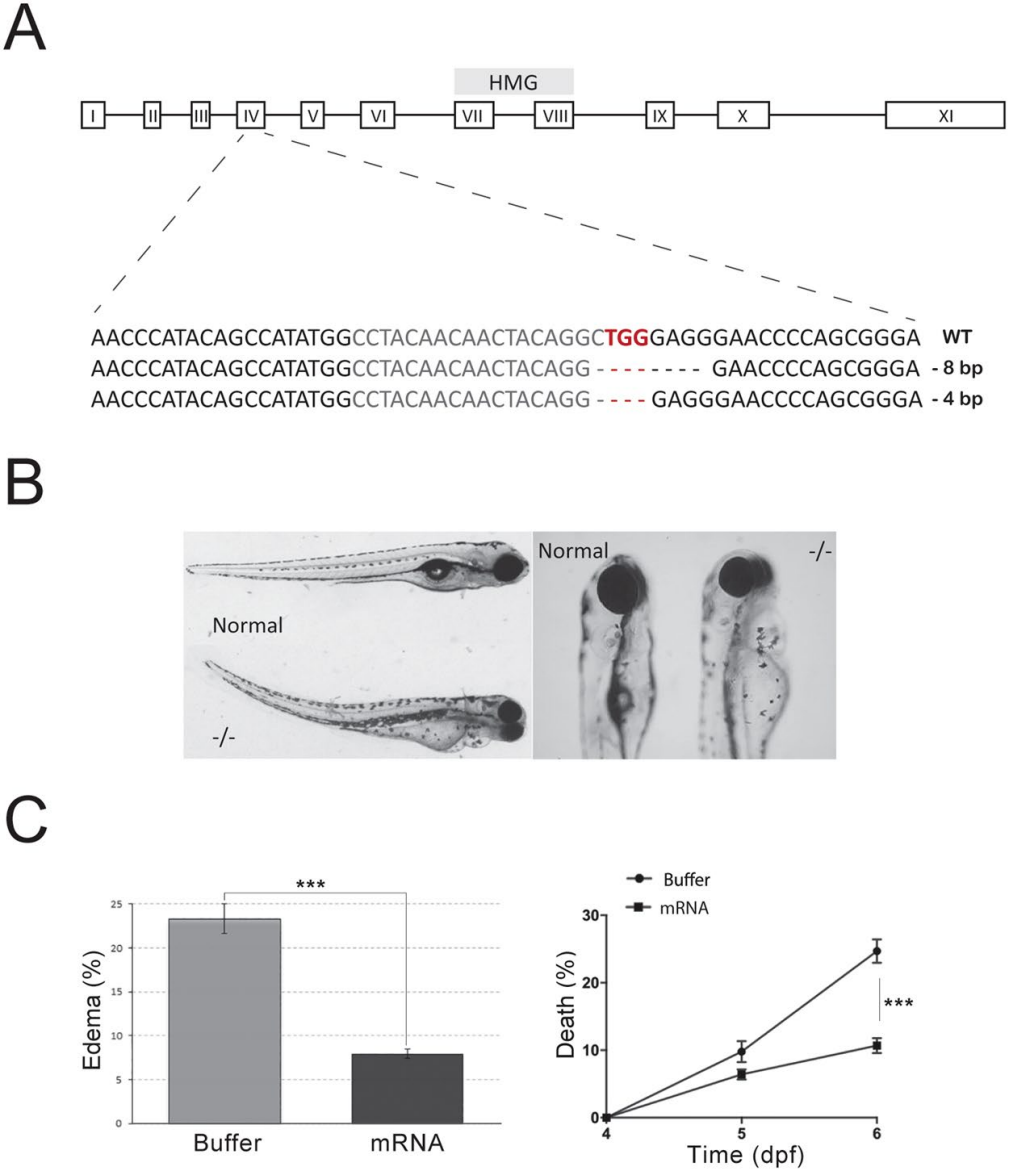
(**A**) Schematic diagram of *smarce1* gene. The DNA-binding high mobility group is shown. CRISPR/Cas9 mediated-deletions were produced in exon IV. The nucleotide sequence of the RNA-guide targeted region is shown in gray with the PAM in red and the deletions are indicated in lines below. (**B**) The body curvature, smaller eyes and cardiac edema are shown. (**C**) Graphs showing rescue experiments with injected *smarce1* mRNA. A significant lower number of edematous embryos (left) and a higher survival rate (right) were determined in groups of the mRNA-injected relative to buffer-injected embryos. Values represent the average of three independent injections of 100 ± 10 embryos per experiment. ***P < 0.001.

A search in the zebrafish GenBank database indicated the presence of one single gene (Gene ID: 322248) in chromosome 3 with homology to the mammalian *SMARCE1* gene. Smarce1 protein shares a sequence identity of 77% and a sequence similarity of 84% to both human and mouse SMARCE1. RT-PCR analysis was performed to verify the expression of the gene along embryonic development. As predicted, *smarce1* transcripts were clearly detected in all embryonic stages tested from 1-cell stage to 5dpf as well as in all organs examined at adult stages (Fig. S1). In addition, whole mount *in situ* hybridization (ISH) revealed that *smarce1* transcripts were ubiquitously distributed in embryos of different developmental stages (Fig. S2). To introduce CRISPR/Cas9-mediated mutations in the *smarce1* gene we designed a guide RNA (gRNA) targeted to exon 4 of the reported genomic sequence. Co-injection of the gRNA with the Cas9 mRNA in single-cell embryos caused frameshift mutations in the *smarce1* gene that created a premature stop codon leading to the production of a short, truncated peptide (53 aa) which lacks all the relevant domains described for Smarce1 protein, such as the HMG domain and a kinesin-like coiled-coiled region that is important for protein-protein interactions (Fig. 1A). Two mutant alleles with four- and eight-nucleotide deletions were identified among F1 fish from which lines were derived and expanded (Fig. 1A). Backcrosses of these alleles yielded offspring, 25% of which had morphological defects. Genotyping of defective embryos confirmed their *smarce1*^−/−^ identity. The most conspicuous morphological defects were detectable at day 3 (dpf) but were fully penetrant at 4 dpf. Alterations included a slight curvature of the tail, noticeable smaller eyes and swelling of the pericardium (Fig. 1B). Larvae were inactive and exhibited a weak response to touch. By 6 dpf cardiac edema became huge and embryos could not move and die. The phenotype was the same for both alleles as well as for heteroallelic combinations. Thus, due to easier genotyping of the 8-base pair deletion, we continued the work with this allele. We confirmed the loss of Smarce1 protein by western blot analysis, where the protein was not detected in the homozygous embryos (Fig. S2), and by ISH, we detected the degradation of the *smarce1* transcripts in the mutant embryos due to non-sense-mediated mRNA decay (Fig. S2). To further demonstrate the specificity of the phenotype, we injected single-cell embryos obtained from *smarce1*^+/−^ incrosses with synthetic *smarce1* mRNA. After 4dpf we quantified the persistence of cardiac edema in the injected embryos and found a significant rescue of this defect in the mRNA-injected compared to buffer-injected embryos (Fig. 1C). By 6 dpf mRNA-injected embryos showed a decrease in mortality from 25% in the buffer-injected embryos, likely corresponding to the homozygous individuals, to 8% in the mRNA-injected embryos (Fig. 1C). These results confirmed that the phenotypes observed were a direct consequence of the loss of the Smarce1 subunit.

### Smarce1 is essential for heart development

**Figure 2:**
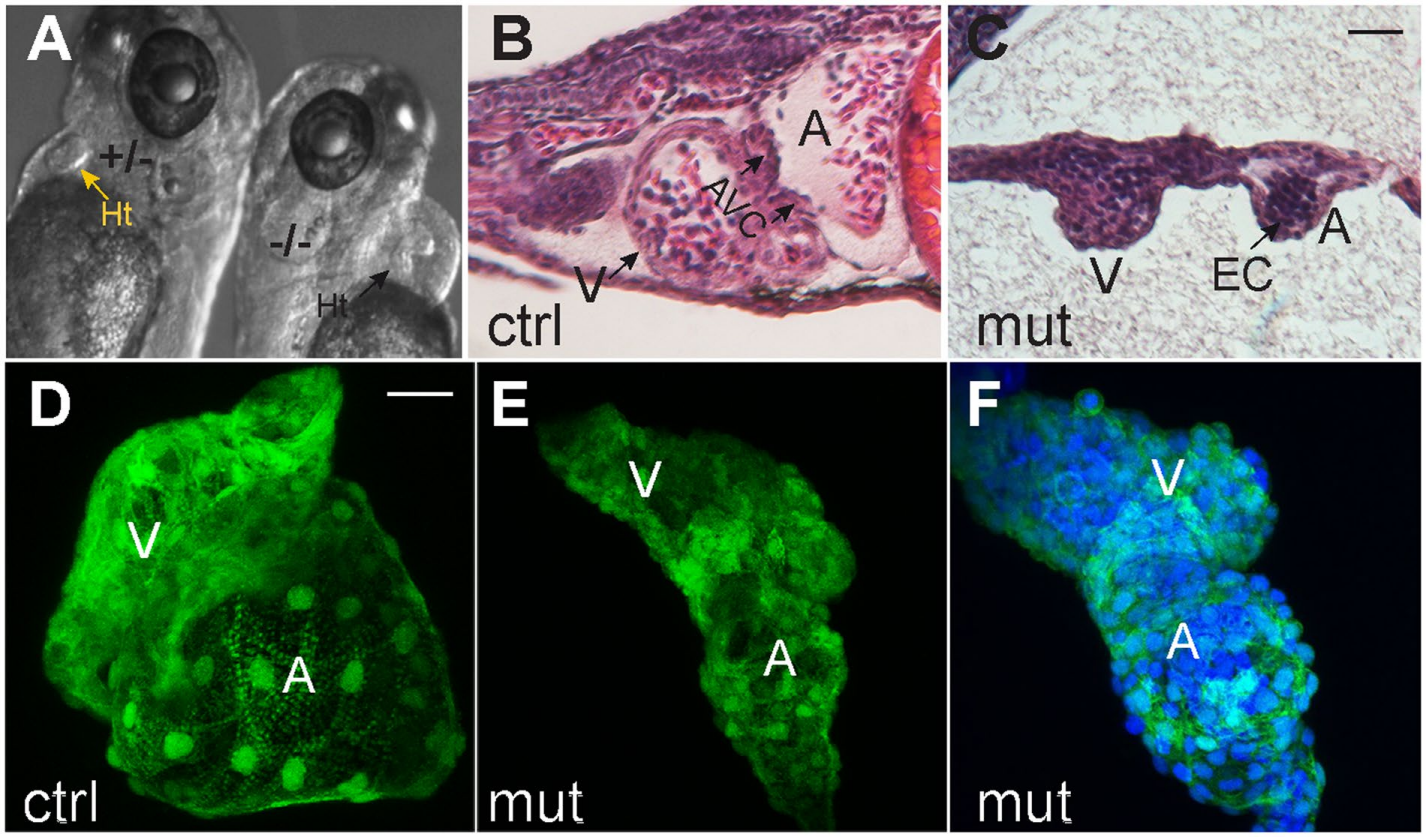
Morphological defects in *smarce1* mutants. (**A**) The heart morphological defects of mutated embryo can be appreciated in comparison with a heart from a normal sibling (arrow) at 72 hpf. B-C. Sagittal sections stained with hematoxylin-eosin from 96 hpf normal (**B**) and mutant (**C**) embryos. (**D–F**) Confocal Z-projections of *cmlc2:GFP/smarce1* normal (**D**) and defective hearts (**E**,**F**) at 72 hpf. In F nuclei were stained with DAPI. A: atria, AVC: atrio-ventricular canal, EC: endocardium, Ht: heart, V: ventricle. Scale bars: 25 μm.

The defects exhibited by the Smarce1 mutants are in general reminiscent of those observed in the two reported *smarca4* mutant alleles, *young* and *brg1*^*s481*^^18,29^. Because of the importance of chromatin remodeling during heart development, and to contribute to the identification of the functional complexes that are active in the embryonic heart, we focused on this phenotypic aspect. Morphological analysis of the heart at 4 dpf indicated the presence of both atrial and ventricular chambers, but with a dysmorphic aspect with the typical tube-like shape described for other zebrafish mutants with heart phenotype like the *heartstrings* mutant in the *tbx5* gene^30^ (Fig. 2A; Movie S1). Myocardial and endocardial cell layers were detected in sagittal sections of mutant hearts, but the formation of the atrio-ventricular canal (AVC) was not evident (Fig. 2B,C). Additionally, in mutant hearts, a cavity inside the lumen could not be appreciated when compared to a section of a control heart (Fig. 2B,C). Images of three-dimensional reconstructions of fixed 3dpf mutant embryos carrying Tg(*cmlc2:egfp*) demonstrated the presence of differentiated cardiomyocytes, but they also revealed tissue disorganization and extreme cell compaction in both chambers, with atria presenting a higher defective aspect (Fig. 2D–F). Video imaging of live embryos indicated that the heart beating is significantly slower (with occasional arrests) and circulation was absent (∼70%) or extremely slow (∼30%) in mutant embryos (Movies S1 and S2).

**Figure 3:**
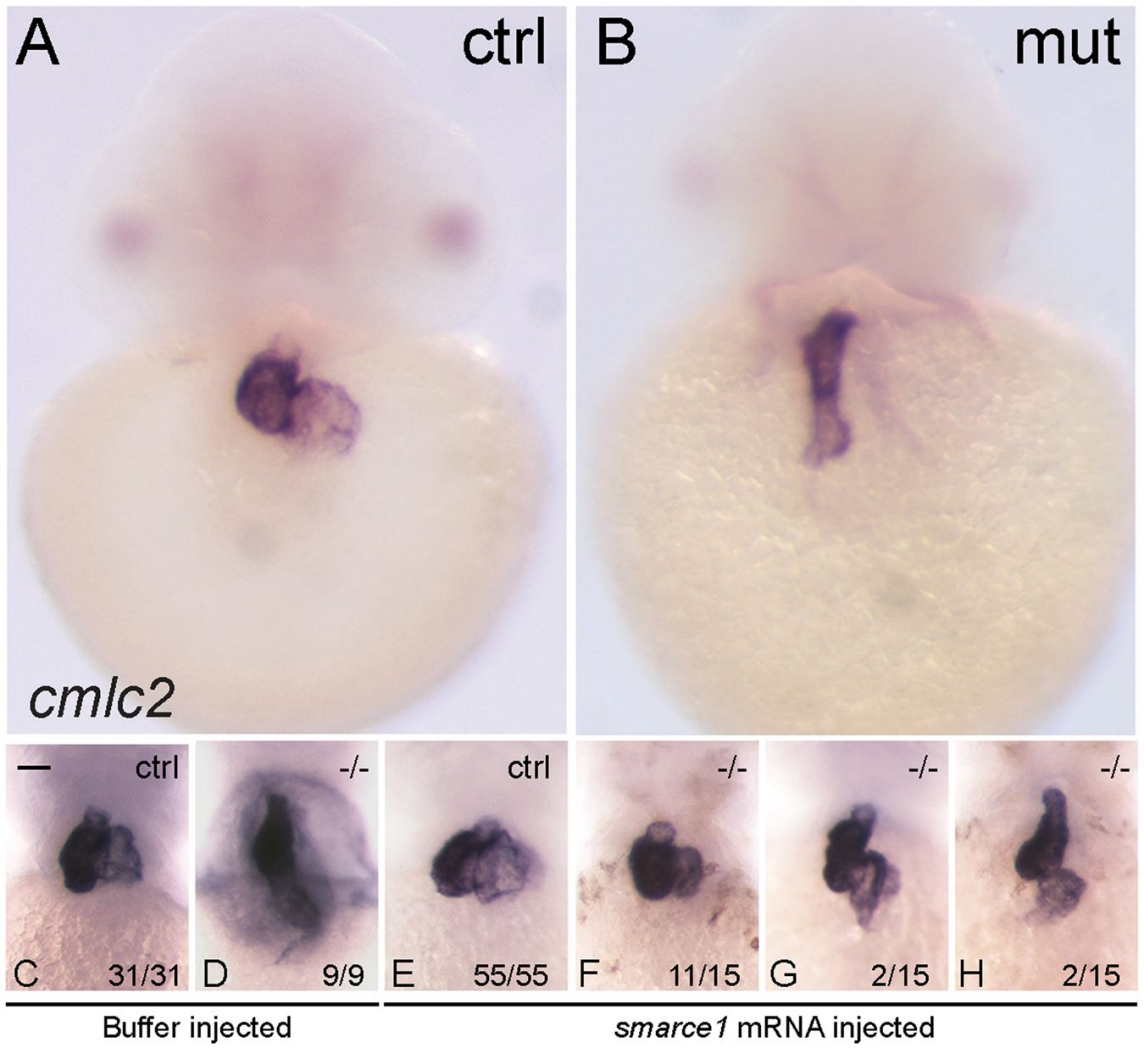
Rescue of the heart looping by *smarce1* mRNA injection in *smarce1* mutant embryos. Whole-mount *in situ* hybridization detects transcripts for the cardiomyocyte marker *cmlc2*. A-B. Embryos at 48 hpf showing a normal heart (**A**) and a *smarce1*^−/−^ heart with a defective looping (**B**). (**C–H**) Analysis of heart looping at 72 hpf. Buffer injected embryos present a normal heart in control siblings (**C**) and a non-looped heart in all the mutants (**D**). (**E–H**) *smarce1* mRNA injected embryos showing the rescue of the heart looping in the mutant embryos. Complete rescue is shown in (**F**); a partial rescue in (**G**); and the failing of rescue in (**H**). Scale bars: 50 μm.

**Figure 4:**
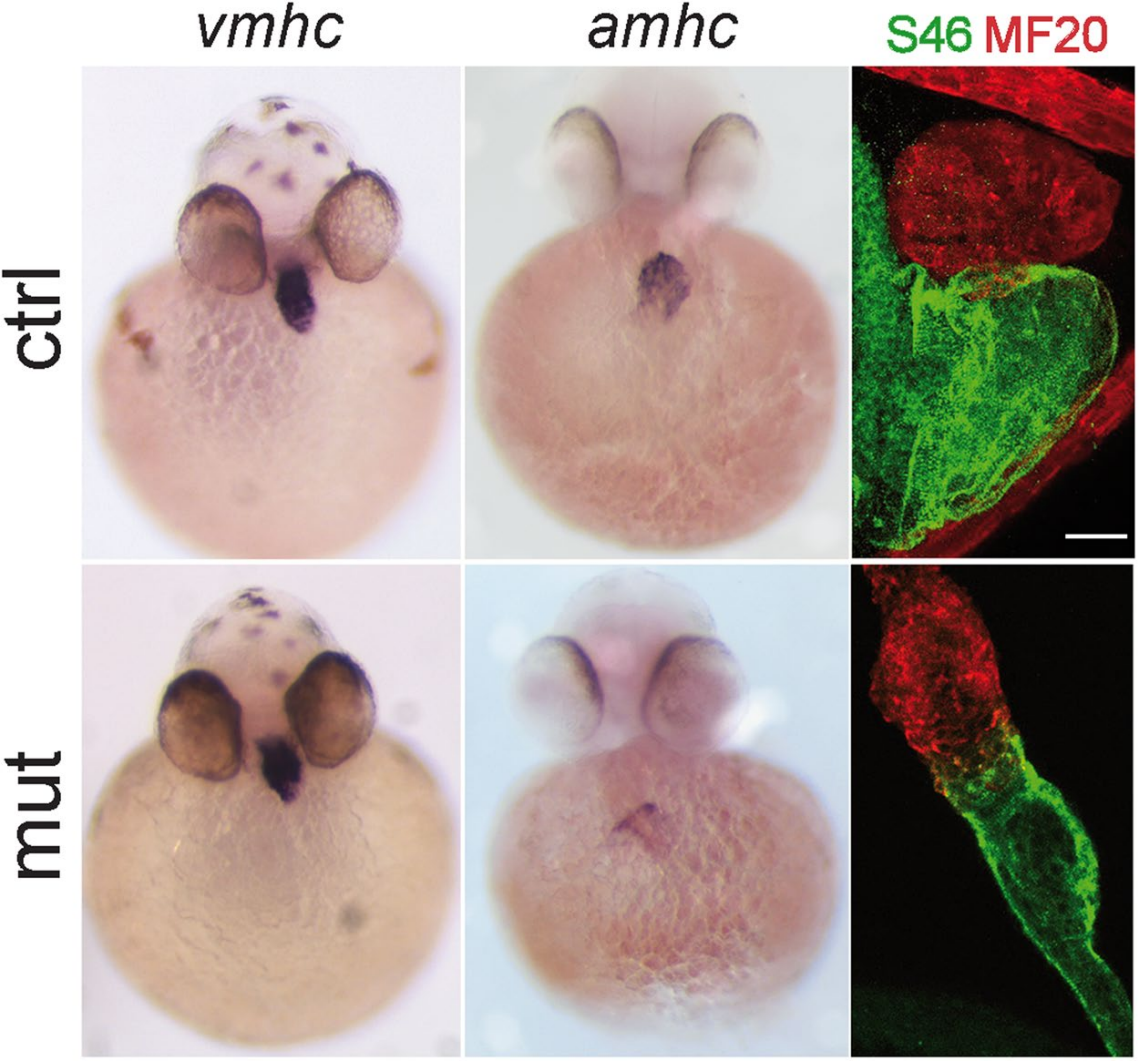
Patterns of expression of atria and ventricle markers in *smarce1* mutants. Chamber expression in *smarce1* heterozygous (upper panel) and homozygous (lower panel) embryos. Left to right: 48 hpf embryos showing the expression of the ventricular cardiomyocyte marker *vmhc*, embryos showing the expression of the atria cardiomyocyte marker *amhc*, 72 hpf embryos stained with MF20 and S46 antibodies to detect the ventricle (red) and atrium (green) in wildtype and mutant embryos. Scale bar: 25 μm.

We performed gene expression analysis of specific marker genes at 48 hpf. ISH with the cardiomyocyte marker *cmlc2* evidenced the failure of the looping process (Fig. 3A,B). In order to evaluate the capacity of Smarce1 to rescue the looping defect, we repeated the assay previously described. Embryos obtained from *smarce1*^+/−^ crosses were injected with the *smarce1* mRNA and probed with *cmlc2* at 72 hpf to reveal the heart aspect. After genotyping, embryos were classified according to their heart morphology (Fig. 3C–H). Hearts from *smarce1*^+/+^ and *smarce1*^+/−^ embryos injected with buffer presented a regular shape in which ventricle and atria were located side by side (Fig. 3C). In contrast, in hearts of *smarce1*^−/−^ embryos, the ventricle was located above the atria (Fig. 3D). In the group of embryos injected with the *smarce1* mRNA, we found that out of 15 *smarce1*^−/−^ embryos, 11 (73%) presented a normal looking heart with side by side chamber organization (Fig. 3F); two (13%) were partially rescued (Fig. 3G); and two (13%) presented a very poor rescued phenotype (Fig. 3H). This result indicated that Smarce1 is indeed capable of restoring the heart shape. Embryos were also probed with *vmhc* and *amhc* to evaluate the differentiation of ventricular and atrial cells respectively (Fig. 4). Both markers were detected in the expected domains, indicating the proper establishment of the cardiac pattern. For a more detailed evaluation of cardiac chamber identity we obtained chamber-specific expression patterns of the Vmhc and Amhc proteins through MF20/S46 immunofluorescence at 72 hpf. We found that the identity of the chambers was not altered; no ventricular to atrial transdifferentiation was detected, and although the morphology of both atria and ventricle was generally defective, the relative proportionality between chambers did not seem to be affected.

### Patterning of the anterior lateral plate mesoderm is normal in the *smarce1* mutants

To evaluate if the differentiation of the mesoderm and the endoderm in general were affected and to identify the developmental stages where cardiac defects originate in the *smarce1* mutants, we determined the expression patterns of mesodermal, endodermal and cardiac markers during epiboly and gastrulation and in the anterior lateral plate mesoderm (ALPM), where the cardiac primordia is contained (Fig. S3). Expression of *no tail (ntl)* in mutant embryos at epiboly and somite stages presented a normal pattern, indicating that mesoderm development is not defective. Similarly, normal expression of *gata5* at 50% epiboly in the germ ring and at 90% epiboly in endodermal cells revealed a proper formation of the endoderm. Next, we examined the expression of the early cardiac markers *gata5, nkx2.5* and *hand2*, which at 6–9 somite stage are expressed in different regions of the ALPM. The expression patterns of these three genes in *smarce1* mutants and wild type siblings were indistinguishable, suggesting that cardiac progenitor cells residing in the lateral and medial ALPM are distributed normally. At 14-somite stage *cmlc2* expression in the mutants demonstrated a normal migration of myocardial cells. Altogether these results show that up to this stage, *smarce1* mutants seem to have a normal heart development.

### Endocardial development in the *smarce1* mutants is abnormal

**Figure 5:**
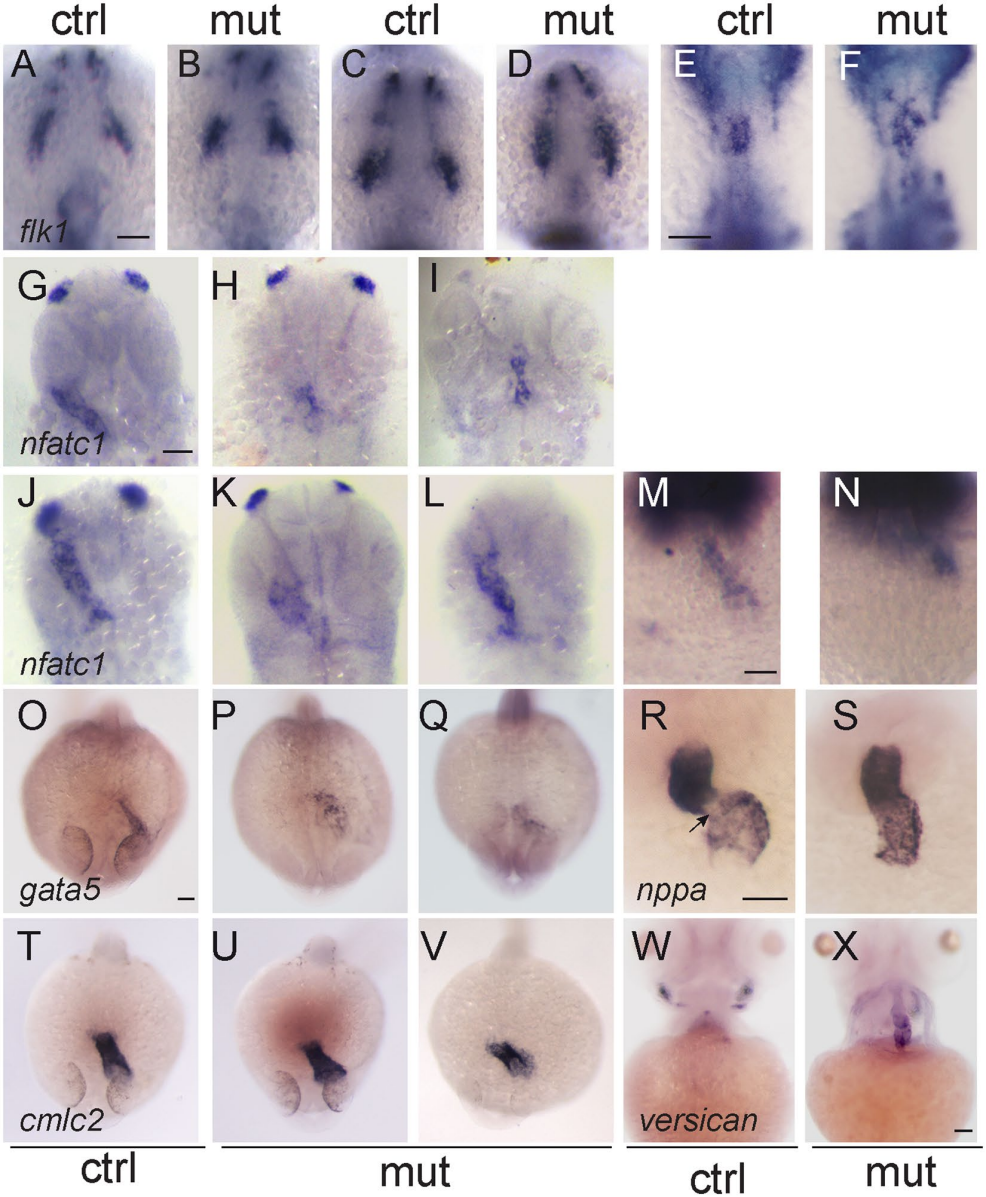
Defects in the endocardium in *smarce1* mutants. Embryos at 9- (**A,B**), 14- (**C,D**) and 20- (**E,F**) somite stage showing the expression the endothelial marker *flk1*. While a normal ALPM pattern is observed in mutant embryos up to 14 somites, at 20 somite stage mutant embryos present an abnormal distribution of endocardial cells. Expression of *nfatc1* in embryos at 26 (G-I), 30 (**J–L**) and 35 hpf (**M,N**) reveals a disorganized pattern of the endocardium. Embryos at 24 hpf showing expression of the endocardial marker *gata5* (**O–Q**) and the myocardial marker *cmlc2* (**T–V**). Expression of *gata5* in the endocardium of the mutant embryos is abnormal and has a lower signal. A dysmorphic pattern for *cmlc2* is detected in one out of six embryos (**V**). R,S. Expression of *nppa* in 48 hpf embryos. An arrow shows the absence of *nppa* at the atrioventricular junction. (**W, X**) Expression of *versican* in embryos at 4 dpf. Scale bars: 50 μm.

Markers associated to endocardial progenitors and vascular cells were also tested. The expression pattern of *flk1* in the ALPM of 9- and 14-somite stage mutants did not show significant defects when compared to that of their siblings, suggesting a proper specification of endocardial progenitors (Fig. 5A–D). At 18-somite stage endocardial progenitors migrating from the rostral ALPM arrive to the midline, where they fuse in the region from which the heart cone will arise; by 20-somite stage an aggregate of endocardial cells with a u-shaped morphology is formed^31^. Expression of *flk1* became apparent in this apex in control embryos. In contrast, in *smarce1* mutants *flk1* expression revealed a disorganized pattern suggesting an abnormal morphogenesis of the endocardium (Fig. 5E,F). *nfatc1* is a molecular marker that is expressed in endocardial, but not vascular endothelial cells. Its earliest expression is apparent by 22 hpf. Expression of the *nfatc1* gene in embryos at 26, 30 and 35 hpf (Fig. 5G–N) confirmed the defective morphology of the endocardium in the *smarce1* mutants. Similarly, expression of *gata5*, which at 24 hpf becomes predominantly endocardial^32^, presented an abnormal pattern in the cardiac region of the mutants (Fig. 5O–Q). Altogether the analysis of these markers led to the conclusion that Smarce1 is required for the correct development of the endocardium in zebrafish embryos. Gene expression analysis at 24 hpf with the *cmlc2* marker indicated that the majority of embryos identified as *smarce1*^−/−^ showed a normal myocardial pattern of expression, reflecting a correct heart tube assembly (Fig. 5T,U). However, one out of six of the mutant embryos, displayed an abnormal pattern (Fig. 5V), consisting in a delayed or incomplete extension of the heart tube and a widening of the signal, which revealed that a dysmorphic heart starts to be detected during assembly and elongation of the heart tube. Given that myocardial-endocardial interactions are central to the process of heart tube assembly^33^, it is possible that the defective endocardium of the *smarce1*^−/−^ mutants fails to organize the cardiomyocytes into an appropriate configuration for a correct shape of the heart tube. This problem could constitute one of the initial causes of heart misshaping in the mutants. We then analyzed the effects of the *smarce1* mutation in the development of the atrioventricular canal (AVC). To this aim we examined *nppa* and *versican* expression at the AV boundary. In wild type embryos *nppa* expression was excluded from the AV boundary while in the mutant hearts, *nppa* mRNA was detected in the entire heart, with no clear exclusion from the AV boundary (Fig. 5R,S). A dramatic change in gene expression was detected for *versican* which marks the AV boundary in normal hearts. At 72 hpf, mutant embryos presented a broad expression of versican in both chambers, with the highest intensity in the lower part of the atrium (Fig. 5W,X). These results show that a normal AV boundary is not formed in the *smarce1* mutants.

### Loss of Smarce1 leads to upregulation of cardiac transcription factors

**Figure 6:**
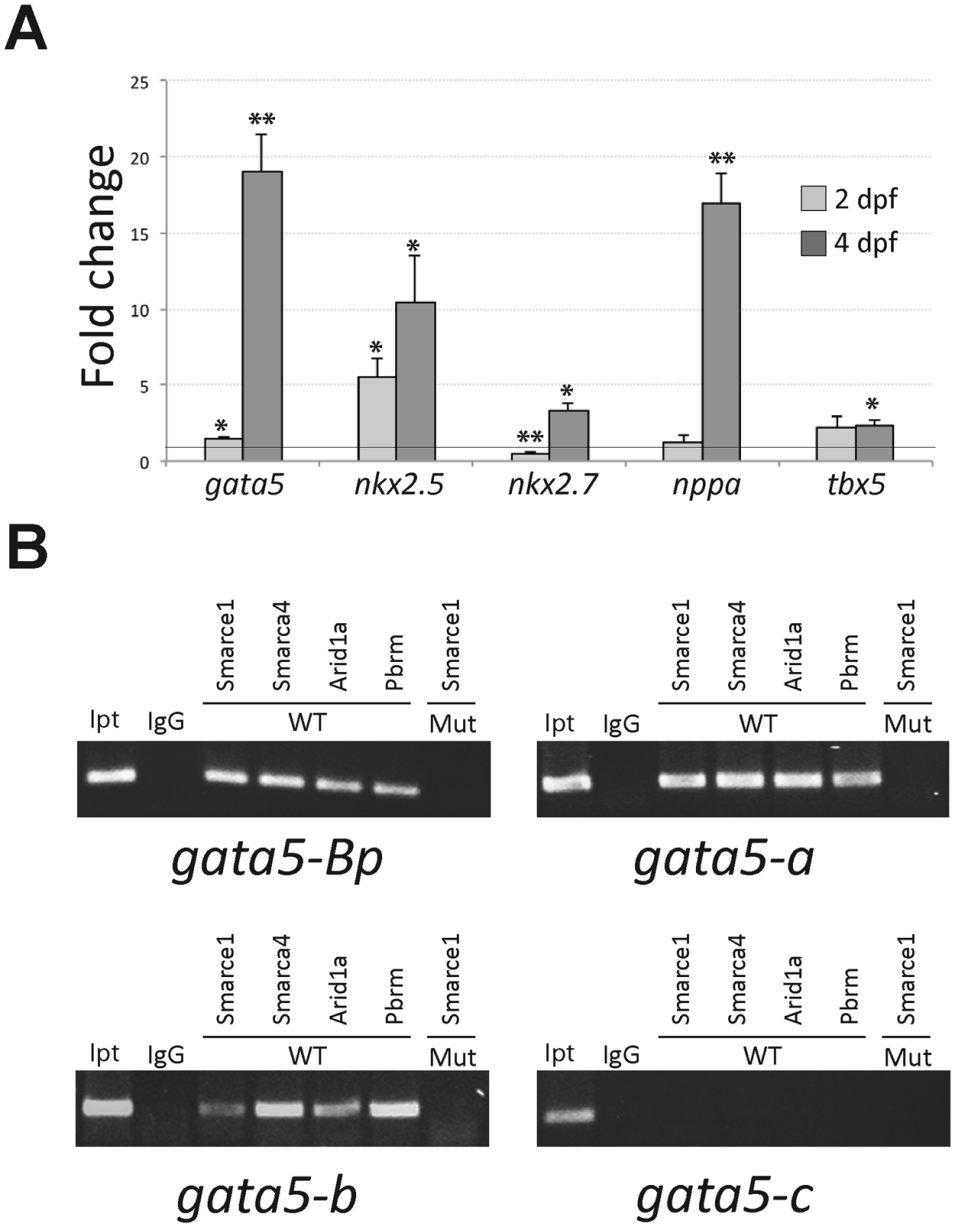
(**A**) Quantitation by QPCR of gene expression of cardiac factors. 2 dpf and 4dpf embryos were genotyped and evaluated. For all genes n ≥ 3. *P < 0.05, **P < 0.01. (**B**) *In vivo* Smarce1-binding sites were detected by chromatin immunoprecipitation using a SMARCE1 antibody. Cropped gel images representing typical results from ChIP assays in: the regulatory module located from the transcription initiation site +1 to −1415, which includes the basic promoter (*gata5*-Bp), the module located from −1469 to −1892 (*gata5*-A), the module located from −7823 to −8213 (*gata5*-B) and the module located from −16805 to −17147 (*gata5*-C). IgG, Smarce1, Smarca4, Arid1a and Pbrm indicate the PCR products from genomic DNA precipitated using the corresponding antibody. WT and Mut indicate the source of genomic DNA. The input is the PCR product from the total input genomic DNA. Full-length gels are presented in Supplementary Figure 3.

We then quantified the expression of cardiac transcription factors. For this purpose, we used 24, 48 and 96 hpf embryos for qPCR. Among the genes quantified at 24 hpf we included *gata5, nkx2.5*, *ntl* and the *smarcd3* genes *a* and *b*. The Smarcd3 subunit was considered because, as already mentioned, it has been identified as the most relevant BAF subunit in a cardio-specific BAF complex and together with Tbx5 and Gata5 is involved in the early induction of cardiomyocytes. We found that none of these genes presented a change in the transcription levels at 24 hpf (Fig. S4). At 48 and 96 hpf we quantified *gata5, nkx2.5, nkx2.7, nppa, tbx5*, and *bmp4*. At 48 hpf *nkx2.5* expression increased five times, while its homologue *nkx2.7* showed a 50% reduction, additionally *gata5* presented a significant 40% increase of expression (Fig. 6A). At 4 dpf, all cardiac factors including *nkx2.7* and with the exception of *bmp4* (not shown), presented a substantial upregulation (Fig. 6A). The TFs that showed the highest increase were *gata5, nkx2.5* and *nppa*, which were elevated between 10 and 20 times. The heart-specific increase of these three factors was validated by ISH at 5 dpf (Fig. S4). For *gata5* additional regions surrounding the yolk showed an increased expression. In order to estimate the upregulation levels specific to the heart of the *smarce1* mutants, we quantified *gata5* in hearts dissected from 4 dpf embryos. We found that *gata5* expression increased six-times compared to control hearts (Fig. S4). Accordingly, gene expression analysis in 4 dpf embryos confirmed the presence of *smarce1* transcripts in the heart, while in mutant embryos no expression was detected. This result showed that upregulation of *gata5* occurs in the heart as well as in other regions of the embryo, suggesting a general participation of Smarce1 in the negative regulation of *gata5*.

In zebrafish *nkx2.5* and *gata5* are among the earliest cardiogenic markers of heart development^34,35^. Nkx2–5 is activated by GATA transcription factors in vertebrates^35,36^. Similarly, Nkx2–5 and GATA factors cooperate to activate transcription of downstream genes such as *nppa*^37,38^. Due to these genetic interactions, the increased transcription levels of these genes in the mutant could be due to the dysregulation of either of them. For this reason, we aimed to analyze the possibility of a direct interaction between Smarce1 and the gene targets identified. To this purpose, we performed chromatin immunoprecipitation using the SMARCE1/BAF57 antibody. Since regulatory regions of the *nkx2* and the *nppa* genes have not yet been reported, we focused on *gata5*. Previously, three functional *cis*-regulatory regions and a basal promoter sequence were identified for the *gata5* gene^39^. Two of these modules (C and B) respectively located approximately 17 and 8 kb upstream of the transcription initiation site (TIS), as well as the promoter sequence, were shown to bind *in vivo* to the Otx2 transcription factor during transcriptional activation. In contrast, the evolutionarily conserved module A located between 1.8 and 1.4 kb upstream of the TIS did not bind to Otx2. Based on these genomic regions we performed chromatin immunoprecipitation using 72 hpf wild type embryos and the SMARCE1 antibody. Of interest, we found that module A and B and the promoter were clearly enriched in this experiment, while module C was not amplified (Figs 6B and S5). The enrichment of these modules was not observed when *smarce1* mutant embryos were tested. Moreover, when antibodies for other BAF subunits including the core subunit Smarca4 and the alternative subunits Arid1a and Pbrm were used, amplification of the same modules was observed in the wild type embryos, whilst no signal was produced from *smarce1* mutant embryos (Fig. S6). This result indicates that Smarce1 is essential for the recruitment of the BAF complex to the *gata5* regulatory regions, and it shows that both the BAF and the PBAF complexes bind to these regions.

In conclusion, our results indicate that Smarce1 activity correlates with the downregulation of *gata5* cardiac transcription factor in 2–4 dpf embryos. Smarce1 binds to *gata5* and recruits the BAF remodeling complex, but further experiments are needed to support a direct function of Smarce1 and the BAF complex in the repression of *gata5*. It also remains to be determined whether Smarce1 binds the other target genes identified, or if the increased transcription levels of these genes in the mutant, reflect the dysregulation of *gata5*.

## Discussion

In this work, we studied the function of the Smarce1 subunit in the development of the zebrafish embryo. To this aim we introduced CRISPR/Cas9 mutations in the *smarce1* gene. SMARCE1 is a constitutive subunit of both of the SWI/SNF complexes BAF and PBAF, and therefore, very early and dramatic defects could have been expected in the loss of function. Nonetheless we found that two different alleles of *smarce1* mutants seemed normal until 3 dpf, likely due to a maternal rescue and probably because some combinations of the BAF complex might remain functional. Detectable morphogenetic defects in the *smarce1* mutants at 4 dpf included smaller eyes, body curvature defects and pericardial edema. The heart defects were analyzed in more detail. These defects were severe in the mutant: circulation was impaired, and heart contractions were arrhythmic, slower and with sporadic arrests. Gene expression analysis at early development demonstrated that cell lineage determination and ALPM patterning were normal in the *smarce1* mutants. Similarly, we demonstrated that atrial and ventricular cardiomyocytes were differentiated and chamber domains were properly determined. However, from 20-somite stage onwards, we detected a disorganized pattern of endocardial cells in the mutant embryos. Additional experiments are needed to determine the origin of the abnormal endocardial development, which could be the result of a deficient differentiation of the endocardial progenitors or a deficient migration towards the cardiac field, among other causes. Previous work has shown the importance of the endocardium for the organization of cardiomyocyte movement during heart tube assembly. Thus, it is likely that the primary defect of the *smarce1* mutants is associated with a deficient regulation by the endocardium of cardiomyocyte movement. This limitation might determine the absence of heart looping that is detected later in the homozygous embryos. The endocardium also is an important component of the AVC^40^. We found evidence indicating that expression at the AV boundary is not correctly restricted in the mutant hearts and in histological sections of the *smarce1* mutants we were unable to detect valve tissue. Therefore, it is possible that endocardial defects also affect the development of the AVC. A more detailed analysis of AVC markers is needed to establish the AVC morphology in the s*marce1* mutants.

We found that in the absence of Smarce1 a number of cardiac transcription factors are over-expressed. For *gata5* we demonstrated that there is a direct interaction between Smarce1 and different *gata5* regulatory regions. This finding indicates that Smarce1 could be involved in the repression of this gene which occurs after 48 hpf, suggesting that down-regulation of some cTFs is required for further aspects of development after their participation in chamber formation. Significantly, we showed that other BAF subunits also bind to the same *gata5* regulatory regions and that their binding is dependent on the presence of Smarce1. This result highlights the importance of Smarce1 as a mediator of the interaction between the *gata5* gene and the BAF complex. A relevant observation is that none of the genes quantified presented transcriptional changes at 24 hpf. This finding indicates that the first cardiac phenotype observed during the formation of the linear heart tube precedes the changes of expression identified here. It is likely that other genes important for the differentiation and migration of endocardial cells are dysregulated at earlier stages in the ALPM. In this regard, it would be interesting to quantify the expression of the transcription factor Scl/Tal1 or of genes associated with the Slit/Robo signaling pathway.

Other examples of repression of cardiac gene transcription by BAF subunits have been previously reported in mice. For instance, in cultured P19 cells during differentiation of cardiomyocytes, ARID1a has been shown to directly repress cardiac gene transcription through physical interactions with the NURD complex^41^. Additionally, in the sinoatrial node of mouse adult hearts ARID1a cooperates with Tbx3 and histone diacetylase 3 to repress the expression of Nkx2–5^42^. It is clear that different combinations of the BAF complex with alternative remodeling activities co-exist in the developing heart. At early stages, mouse SMARCD3 interacts with GATA4 and TBX5 transcription factors, and the three of them together cause the recruitment of the BAF complex to occupy and open cardiogenic loci in chromatin such as the early enhancer of Nkx2–5. It is possible that other BAF combinations promote the closing of chromatin at later stages. Although Smarce1 is present in all putative combinations, we speculate that it is an important subunit in the complex when repressive activities are required. Significantly, Smarce1 itself has been shown to participate as a repressor in different contexts. One of the best described is the repression of CD4 in mouse T cells, where SMARCE1 participates in the remodeling of chromatin at the CD4 silencer thereby enabling the repressor Runx1 to access the silencer and repress CD4^13,43^. In human cells SMARCE1 and SMARCA4 associate with the methyl-CpG-binding protein MeCP2, and they assemble together in the chromatin of methylated genes and cooperate to silence genes such as the fragile X syndrome FMR1 gene^27^. Interactions of SMARCE1 with repressor complexes such as co-REST in non-neuronal cells have also been reported.

In conclusion, in this work we found that the loss of Smarce1 does not affect the initial steps of development. At later stages, Smarce1 is critical for endocardial development, heart tube formation and heart looping and posteriorly, it functions to repress cardiac gene transcription. This finding is relevant to human genetics as *Smarce1* gene in humans has been associated with the Coffin-Siris syndrome, a disease that includes congenital heart problems. Clearly, other essential roles for Smarce1 are inferred in addition to the one described in cardiac development.

## Methods

### Fish maintenance and strains

A zebrafish (*Danio rerio*) AB-TU-WIK hybrid line was used. The embryos were obtained by natural crosses and raised at 28 °C based on standard procedures^44^. Staging was performed according to the Kimmel system^45^. Zebrafish were handled in compliance with local animal welfare regulations and all experimental protocols were approved by the Comité de ética (Instituto de Biotecnología, UNAM).

### CRISPR/Cas9-mediated mutations and genotyping

CRISPR/Cas9 target sites were designed using an online tool ZiFiT Targeter software (http://zifit.partners.org/ZiFiT). The *smarce1* genomic target sequence is 5′CCTACAACAACTACAGGCTGG3′, located at exon 4. The primers 5′AAACGCCTGTAGTTGTTGTAGG3′ and 5′TAGGCCTACAACAACTAGAGGC3′ were annealed and cloned into the pDR274 plasmid^46^ used BsaI. sgRNA was synthesized using T7 RNA polymerase (Roche). AmpliCap SP6 High Yield Message (CellScript) was used for the Cas9 mRNA synthesis using the pCS2-nls-zCas9-nls plasmid^47^. One-cell stage embryos were injected directly into the cell with ~13 ng/μl of sgRNA, and ~40 ng/μl of Cas9 mRNA diluted in 100 mM KCl. Embryos injected only with Cas9 were used as controls.

The targeted genomic locus was amplified with Phusion High-Fidelity DNA Polymerase (Thermo) from single embryos or larvae using the following primers: 5′CATCGCGTACCCACATCCAC3′ and 5′TCCTCTCGGCTGGGCTTTTA3′, with annealing to sites ~302 and ~96 bp upstream and downstream from the target site respectively. The PCR product was cloned into a TOPO plasmid (Invitrogen) for sequencing. For genotyping, the primers 5′TCTGGCCTACAACAACTACA3′ and 5′TCCTCTCGGCTGGGCTTTTA3′ were used, flanking the mutated site, to amplify a 121 bp fragment, and PCR products were separated by the heteroduplex formation assay on 14% polyacrylamide gels, as described in^48^.

### mRNA synthesis and rescue experiments

The pCS2+ plasmid containing the cDNA of *smarce1* was used for mRNA synthesis with the mMessage mMachine SP6 kit (Ambion). Previous to the rescue assays 100, 400 and 800 pg of *smarce1* mRNA were separately injected into wild type embryos to generate a dosage-response curve. The optimal amount of mRNA that did not produce a significant number of dead or defective embryos in comparison to buffer-injected controls was 400 pg. For the subsequent experiments one-cell stage embryos obtained from heterozygous crosses were injected with 400 pg of *smarce1* mRNA.

### Histology

Embryo were fixed overnight at 4 °C in Bouin´s solution and then dehydrated by a series of graded ethanol. Samples were embedded with paraffin after xylene. Tissue sections of 10 μm were cut and stained in hematoxylin-eosin based on a reported protocol^49^. Subsequently, sections were mounted and photographed on a Leica DMLB microscope equipped with an AxioCam MR5 (Zeiss) camera.

### *In situ* hybridization

The RNA *in situ* hybridization using DIG-labeled antisense RNA probes was performed from reported standard protocols^50^. The plasmids used for *in situ* probe synthesis were previously described and generously donated as follows: *nkx2.5, cmlc2/myl7*, *amhc* and *vmhc* by Ian Scott^25^, *gata5, versican, nppa* and *hand2* by Deborah Yelon^51^*, flk1* by Stephanie Woo^31^ and *nfatc1* by Saulius Sumanas^52^. For synthesis of the *baf57 in situ* probe the following primers were designed: 5′CACAACTCTCCAGCCTACCTT3′ and 5′TTGTGGCTGGGTGGGCA3′.

### Microscopy and analysis

For live imaging, embryos were anaesthetized using 0.016% tricaine (Sigma). Both live and fixed embryos were mounted in 0.6% low-melting agarose. Fluorescent image acquisition was performed using a Zeiss LSM exciter on an Axio Observer confocal microscope. Confocal stacks were processed for maximum intensity projections with Zeiss ZEN2009 software or ImageJ software. Images were adjusted for brightness and contrast using ImageJ. 3D reconstructions and movies were assembled using ImageJ.

### Immunofluorescence

Whole-mount immunofluorescence was performed as previously described^51^ using the monoclonal antibodies MF20 and S46 (Developmental Studies Hybridoma Bank). The secondary reagents goat anti-mouse IgG1–FITC (fluorescein isothiocyanate) and goat anti-mouse IgG2b–TRITC (tetramethylrhodamine isothiocyanate) (SouthernBiotech) recognize S46 and MF20, respectively. MF20 or S46 positive signal were observed by confocal microscopy.

### Real-time PCR analysis

For standard experiments, groups of approximately 20 embryos were collected and transferred to the Trizol Reagent (Ambion) for RNA extraction following the manufacturer’s instructions. When isolated hearts were used, 15 hearts were dissected with a needle as previously described^53^ and transferred to Trizol. After DNAse I (Thermo) treatment,1 μg was used for reverse transcription with M-MLV (Invitrogen) with oligo dT. cDNA was diluted 1:40 in 20 μl quintuplicate reactions. The Maxima SYBR Green Reagent (Thermo) was used for qPCR in a Light Cycler 480 (Roche), using the following program: 95 °C, 5 min; (95 °C, 15 s; 58 °C, 20 s; 72 °C, 30 s-single quantification at this step-) ×40 cycles; and a melting curve from 72 to 95 °C holding during 5 s each 0.5 °C was performed. A relative quantification with the Light Cycler 480 software was performed with at least three of the five replicates that displayed similar reaction curves, after normalizing to the expression level of the elongation factor 1 alpha *(ef1alpha*) and using a second derivative maximum method. *myl7, amhc, vmhc, nkx2.5, tbx5* and *gata5* primers were reported in^54^, *ntl* in^55^.

### Western blot

Lysates were prepared using the Active Motif kit protocol. Proteins were quantified by Bradford assay (Bio-Rad) according to the manufacturer’s instructions. Proteins were separated by sodium dodecyl sulfate–polyacrylamide gel electrophoresis (SDS–PAGE). The gels were blotted onto a Nitrocellulose Membrane (Bio-Rad) and blocked with 5% non-fat dry milk and reacted with the appropriate antibodies for BAF subunits: anti-BAF57/SMARCE1 antibody (Abcam ab131328) and anti-SNF5/SMARCB1 antibody (Abcam ab126734), which are rabbit monoclonal against the human subunits and are known to cross-react with zebrafish Smarce1 and Smarcb1 respectively, all membranes were incubated with anti-ERK2 antibody (Santa Cruz Biotechnology, USA; Cat. Nr. sc-153) to confirm equal protein loading. The blots were posteriorly incubated with horseradish peroxide HRP-conjugated anti-mouse IgG or HRP-conjugated anti-rabbit IgG (Santa Cruz Biotechnology). Chemiluminescence was detected with enhanced chemiluminescence (ECL) western blot detection kits (Thermo Scientific Pierce).

### Chromatin immunoprecipitation (ChIP)

For immunoprecipitation, 100 zebrafish larvae at 4 dpf were used. Chromatin immunoprecipitation was performed as described previously^56^ with few modifications. First, sonication was used to shear the DNA with an ultrasonic disintegrator (model cv 334 Soniprep 150 MSE) set at medium, with 30 s ON and 30 s OFF for a total of 6 cycles. DNA was kept on ice during the sonication. The sizes of the DNA fragments ranged from 300 to 500 base pairs and were validated by gel electrophoresis. Immunoprecipitation (IP) was performed using 1.5 μg (15 μl × 0.1 μg/μl) of BAF57/SMARCE1, BRG1 (Abcam ab110641), ARID1A (NB100-55334 Novus Biological), BAF180/PBRM1 (ARP39332_P050 Aviva Systems Biology) antibodies (or IgG) bound to 20 μl of dynabeads in 80 μl of RIPA buffer at 4 °C, and the beads were added to 100 μl of chromatin. The products were reverse-crosslinked, and the DNA was eluted with 50 μl of elution buffer and then purified by phenol:chloroform:isoamylalcohol extraction. The PCR primers gata5-chip-A gata5-chip-B gata5-chip-C used to check the chromatin immunoprecipitation results were reported in^39^, and for gata5-chip-Bp, the following primers were designed: 5′ACCTCGACGGCGATATTCAA3′ and 5′ATCATCCGCGGGAATCAAGC3′. Forty PCR cycles were performed to check for enrichment.

## Electronic supplementary material

Supplementary figures

Movie 1

Movie 2

## References

[1] Wang WD (1996). Purification and biochemical heterogeneity of the mammalian SWI-SNF complex. Embo Journal.

[2] Son EY, Crabtree GR (2014). The Role of BAF (mSWI/SNF) Complexes in Mammalian Neural Development. American Journal of Medical Genetics Part C-Seminars in Medical Genetics.

[3] Ho L (2009). An embryonic stem cell chromatin remodeling complex, esBAF, is essential for embryonic stem cell self-renewal and pluripotency. Proceedings of the National Academy of Sciences of the United States of America.

[4] Aigner S, Denli AM, Gage FH (2007). A novel model for an older remodeler: The BAF swap in neurogenesis. Neuron.

[5] Wu JI, Lessard J, Crabtree GR (2009). Understanding the Words of Chromatin Regulation. Cell.

[6] Lomeli H, Castillo-Robles J (2016). The developmental and pathogenic roles of BAF57, a special subunit of the BAF chromatin-remodeling complex. Febs Letters.

[7] Kadoch C., Crabtree G. R. (2015). Mammalian SWI/SNF chromatin remodeling complexes and cancer: Mechanistic insights gained from human genomics. Science Advances.

[8] Hargreaves D, Crabtree G (2011). ATP-dependent chromatin remodeling: genetics, genomics and mechanisms. Cell Research.

[9] Yan ZJ (2005). PBAF chromatin-remodeling complex requires a novel specificity subunit, BAF200, to regulate expression of selective interferon-responsive genes. Genes & Development.

[10] Wang WD (1998). Architectural DNA binding by a high-mobility-group/kinesin-like subunit in mammalian SWI/SNF-related complexes. Proceedings of the National Academy of Sciences of the United States of America.

[11] Link KA (2005). BAF57 governs androgen receptor action and androgen-dependent proliferation through SWI/SNF. Molecular and Cellular Biology.

[12] Garcia-Pedrero JM, Kiskinis E, Parker MG, Belandia B (2006). The SWI/SNF chromatin remodeling subunit BAF57 is a critical regulator of estrogen receptor function in breast cancer cells. Journal of Biological Chemistry.

[13] Chi TH (2002). Reciprocal regulation of CD4/CD8 expression by SWI/SNF-like BAF complexes. Nature.

[14] Battaglioli E (2002). REST repression of neuronal genes requires components of the hSWI-SNF complex. Journal of Biological Chemistry.

[15] Smith MJ (2013). Loss-of-function mutations in SMARCE1 cause an inherited disorder of multiple spinal meningiomas. Nature Genetics.

[16] Smith MJ (2014). Germline SMARCE1 mutations predispose to both spinal and cranial clear cell meningiomas. Journal of Pathology.

[17] Tsurusaki Y (2014). Coffin-Siris syndrome is a SWI/SNF complex disorder. Clinical Genetics.

[18] Takeuchi, J. K. *et al*. Chromatin remodelling complex dosage modulates transcription factor function in heart development. *Nature Communications* **2**, 10.1038/ncomms1187 (2011). 10.1038/ncomms1187 PMC3096875 21304516

[19] Lickert H (2004). Baf60c is essential for function of BAF chromatin remodelling complexes in heart development. Nature.

[20] Takeuchi JK, Bruneau BG (2009). Directed transdifferentiation of mouse mesoderm to heart tissue by defined factors. Nature.

[21] Takeuchi JK (2007). Baf60c is a nuclear Notch signaling component required for the establishment of left-right asymmetry. Proceedings of the National Academy of Sciences of the United States of America.

[22] Huang XL, Gao XL, Diaz-Trelles R, Ruiz-Lozano P, Wang Z (2008). Coronary development is regulated by ATP-dependent SWI/SNF chromatin remodeling component BAF180. Developmental Biology.

[23] Wang Z (2004). Polybromo protein BAF180 functions in mammalian cardiac chamber maturation. Genes & Development.

[24] Scott IC, Bruneau BG (2012). Life Before Nkx2.5: Cardiovascular Progenitor Cells: Embryonic Origins And Development. Heart Development.

[25] Lou X, Deshwar AR, Crump JG, Scott IC (2011). Smarcd3b and Gata5 promote a cardiac progenitor fate in the zebrafish embryo. Development.

[26] Singh AP, Archer TK (2009). SWI/SNF-BAF250A is remodeling chromatin in early embryogenesis and heart development. Developmental Biology.

[27] Harikrishnan KN (2005). Brahma links the SWI/SNF chromatin-remodeling complex with MeCP2-dependent transcriptional silencing. Nature Genetics.

[28] Faralli H (2011). Teashirt-3, a Novel Regulator of Muscle Differentiation, Associates with BRG1-associated Factor 57 (BAF57) to Inhibit Myogenin Gene Expression. Journal of Biological Chemistry.

[29] Gregg RG, Willer GB, Fadool JM, Dowling JE, Link BA (2003). Positional cloning of the young mutation identifies an essential role for the Brahma chromatin remodeling complex in mediating retinal cell differentiation. Proceedings of the National Academy of Sciences of the United States of America.

[30] Garrity DM, Childs S, Fishman MC (2002). The heartstrings mutation in zebrafish causes heart/fin Tbx5 deficiency syndrome. Development.

[31] Bussmann J, Bakkers J, Schulte-Merker S (2007). Early endocardial morphogenesis requires Scl/Tal1. PLoS Genet.

[32] Reiter J (1999). Gata5 is required for the development of the heart and endoderm in zebrafish. Genes & Development.

[33] Holtzman N, Schoenebeck J, Tsai H, Yelon D (2007). Endocardium is necessary for cardiomyocyte movement during heart tube assembly. Development.

[34] Targoff KL, Schell T, Yelon D (2008). Nkx genes regulate heart tube extension and exert differential effects on ventricular and atrial cell number. Dev Biol.

[35] Peterkin T, Gibson A (2003). & Patient, R. GATA-6 maintains BMP-4 and Nkx2 expression during cardiomyocyte precursor maturation. Embo Journal.

[36] Brown C (2004). The cardiac determination factor, Nkx2-5, is activated by mutual cofactors GATA-4 and Smad1/4 via a novel upstream enhancer. Journal of Biological Chemistry.

[37] Horsthuis T (2008). Distinct regulation of developmental and heart disease-induced atrial natriuretic factor expression by two separate distal sequences. Circulation Research.

[38] Sergeeva I, Christoffels V (2013). Regulation of expression of atrial and brain natriuretic peptide, biomarkers for heart development and disease. Biochimica Et Biophysica Acta-Molecular Basis of Disease.

[39] Tseng W-F, Jang T-H, Huang C-B, Yuh C-H (2011). An evolutionarily conserved kernel ofgata5, gata6, otx2 and prdm1a operates in the formation of endoderm in zebrafish. Developmental Biology.

[40] Haack T, Abdelilah-Seyfried S (2016). The force within: endocardial development, mechanotransduction and signalling during cardiac morphogenesis. Development.

[41] Singh AP, Archer TK (2014). Analysis of the SWI/SNF chromatin-remodeling complex during early heart development and BAF250a repression cardiac gene transcription during P19 cell differentiation. Nucleic Acids Research.

[42] Wu M (2014). Baf250a orchestrates an epigenetic pathway to repress the Nkx2.5-directed contractile cardiomyocyte program in the sinoatrial node. Cell Research.

[43] Wan M (2009). Molecular basis of CD4 repression by the Swi/Snf-like BAF chromatin remodeling complex. Eur J Immunol.

[44] Westerfield, M. *The zebrafish book: a guide for the laboratory use of zebrafish (Danio rerio)*. (Univ. of Oregon Press, 2000).

[45] Kimmel CB, Ballard WW, Kimmel SR, Ullmann B, Schilling TF (1995). Stages of embryonic development of the zebrafish. Dev Dyn.

[46] Hwang W (2013). Efficient genome editing in zebrafish using a CRISPR-Cas system. Nature Biotechnology.

[47] Jao L, Wente S, Chen W (2013). Efficient multiplex biallelic zebrafish genome editing using a CRISPR nuclease system. Proceedings of the National Academy of Sciences of the United States of America.

[48] Chen J (2012). Efficient Detection, Quantification and Enrichment of Subtle Allelic Alterations. DNA Research.

[49] Cardiff RD, Miller CH, Munn RJ (2014). Manual hematoxylin and eosin staining of mouse tissue sections. Cold Spring Harb Protoc.

[50] Thisse C, Thisse B (2008). High-resolution *in situ* hybridization to whole-mount zebrafish embryos. Nature Protocols.

[51] Yelon D, Horne S, Stainier D (1999). Restricted expression of cardiac myosin genes reveals regulated aspects of heart tube assembly in zebrafish. Developmental Biology.

[52] Palencia-Desai S (2015). Myocardium and BMP signaling are required for endocardial differentiation. Development.

[53] Yang, J. & Xu, X. Immunostaining of dissected zebrafish embryonic heart. *J Vis Exp*, e3510, 10.3791/3510 (2012). 10.3791/3510 PMC3369776 22258109

[54] Dohn TE, Waxman JS (2012). Distinct phases of Wnt/β-catenin signaling direct cardiomyocyte formation in zebrafish. Dev Biol.

[55] Su J, Zhu Z, Wang Y, Xiong F, Zou J (2008). The cytomegalovirus promoter-driven short hairpin RNA constructs mediate effective RNA interference in zebrafish *in vivo*. Marine Biotechnology.

[56] Lindeman L, Vogt-Kielland L, Alestrom P, Collas P (2009). Fish’n ChIPs: Chromatin Immunoprecipitation in the Zebrafish Embryo. Chromatin Immunoprecipitation Assays: Methods and Protocols.

