# Supplementary material for: smarce1 mutants have a defective endocardium and an increased expression of cardiac transcription factors in zebrafish
Source: Sci Rep. 2018 Oct 18;8:15369. doi: 10.1038/s41598-018-33746-8 (PMC6194089; doi:10.1038/s41598-018-33746-8)
Supplement: Supplementary file 1 — Supplementary figures [file 41598_2018_33746_MOESM1_ESM.pdf]

# *smarce1* mutants have a defective endocardium and an increased expression of cardiac transcription factors in zebrafish

Jorge Castillo-Robles, Laura Ramírez, Herman P. Spaink and Hilda Lomelí

Supplementary figures.

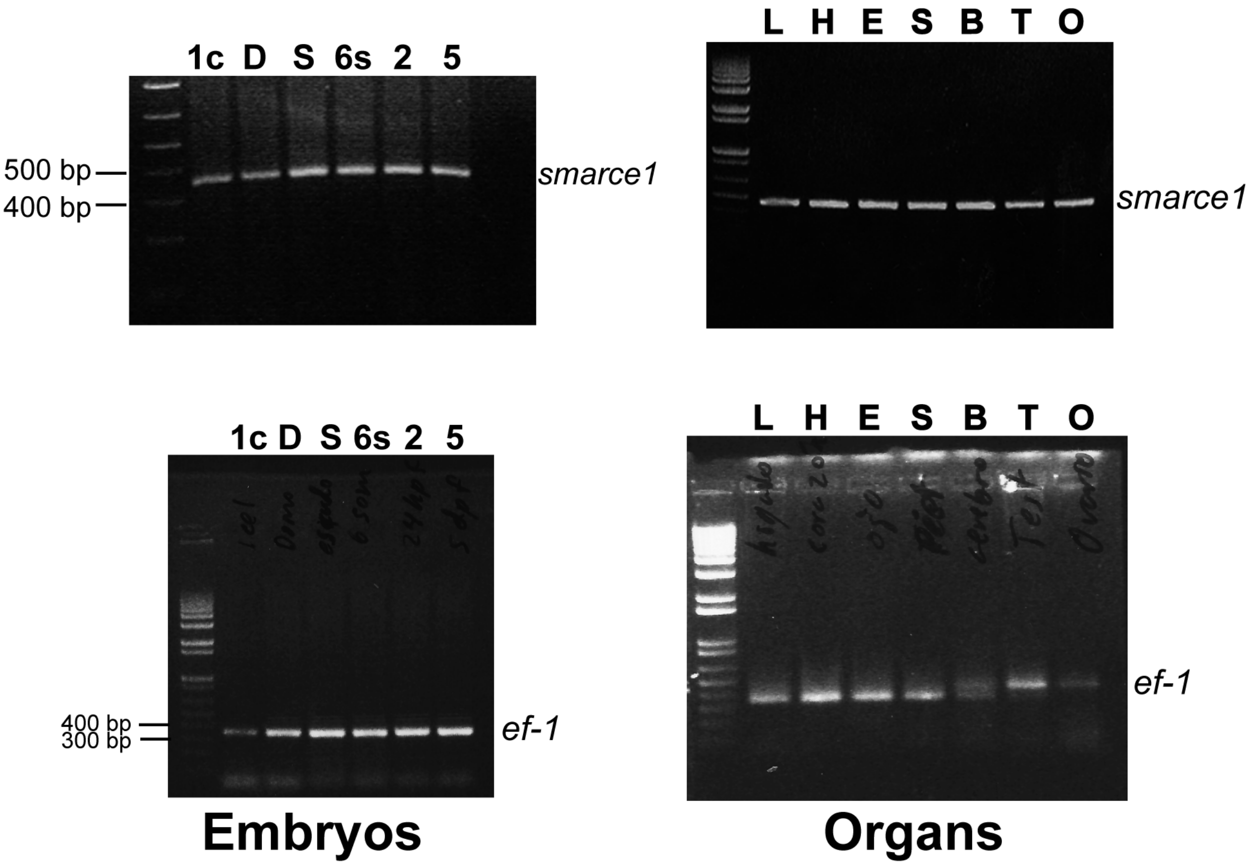


Supplementary figure 1.

Full-length gel images showing expression of *smarce1* detected by RT-PCR. Expected length for amplicons: *smarce1*, 472 bp; *elongation factor-1*, 358 bp. Left panels, expression in development: 1-cell (1c), dome (D), shield (S), six somites (6s), 2 dpf (2) and 5 dpf (5) embryos. Right panels, expression in adult organs: liver (L), heart (H) eye (E) skin (S) brain (B) testis (T), ovary (O).

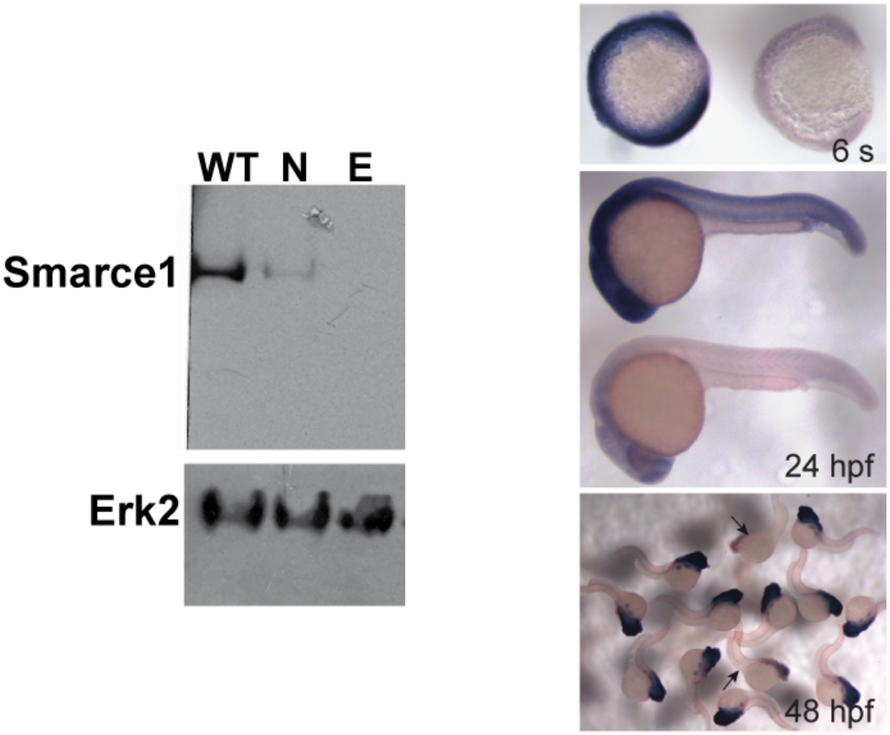


Supplementary figure 2.

Left panel. Western blot with SMARCE1 antibody from 4 dpf wild type embryos (WT), normal looking embryos derived from a smarce1$^{+/-}$ intercross (N) and their edematous siblings (E). Right panel. Gene expression pattern of *smarce1* in progenies derived from a *smarce1*$^{+/-}$ intercross at the indicated stages (s = somites). Embryos with fade signal (arrows) were determined to be *smarce1*$^{-/-}$ homozygous.

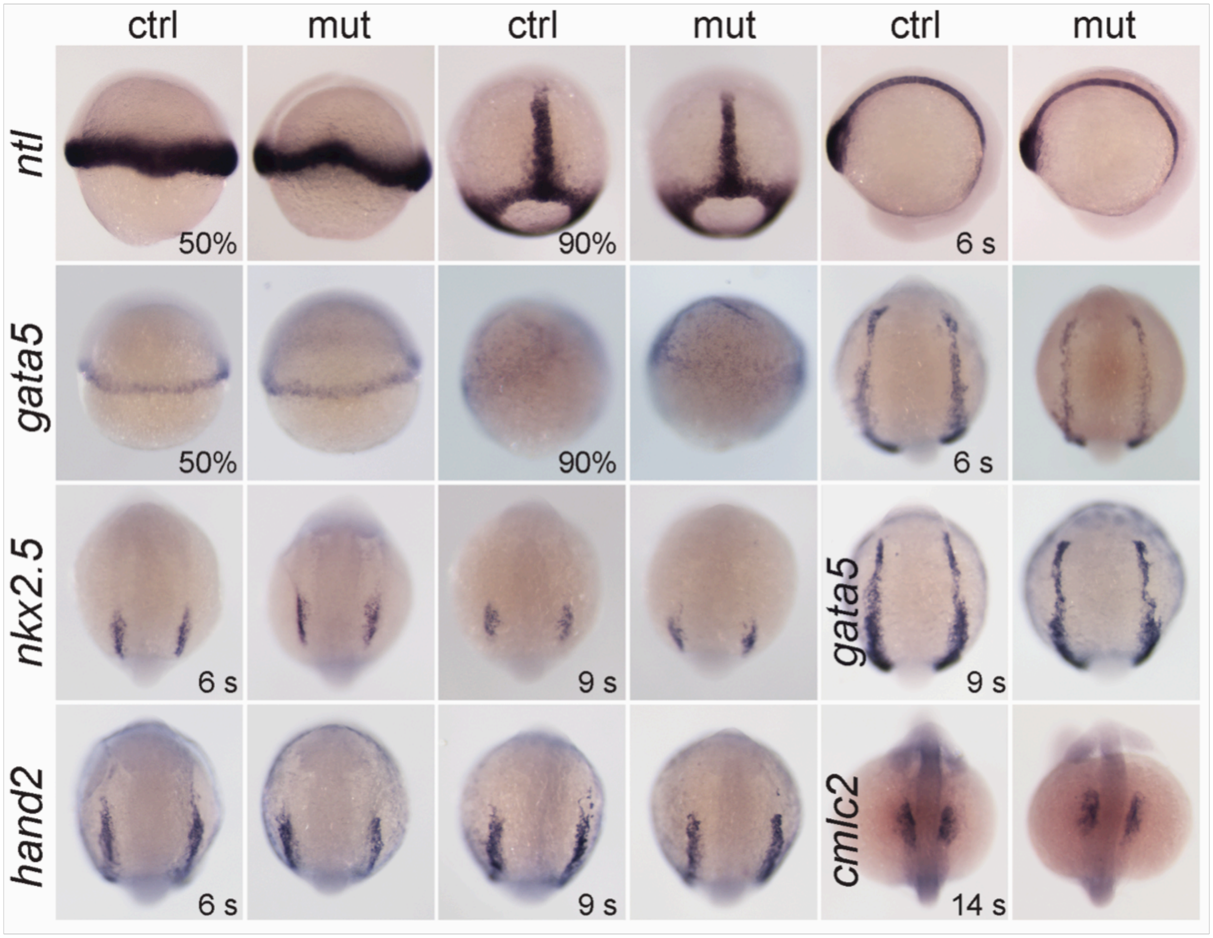


Supplementary figure 3.

Gene expression patterns in control and *smarce1*$^{-/-}$ embryos for the indicated transcripts at the indicated stages (s = somites). Embryos at 50%, and 90% epiboly, dorsal views with anterior to the top. Embryos at 6-somite stage for *ntl*, lateral views. Embryos at 6-, 9- and 14-somite stage for *gata5*, *hand2*, *nkx2.5* and *cmlc2*, dorsal views with anterior to the top.

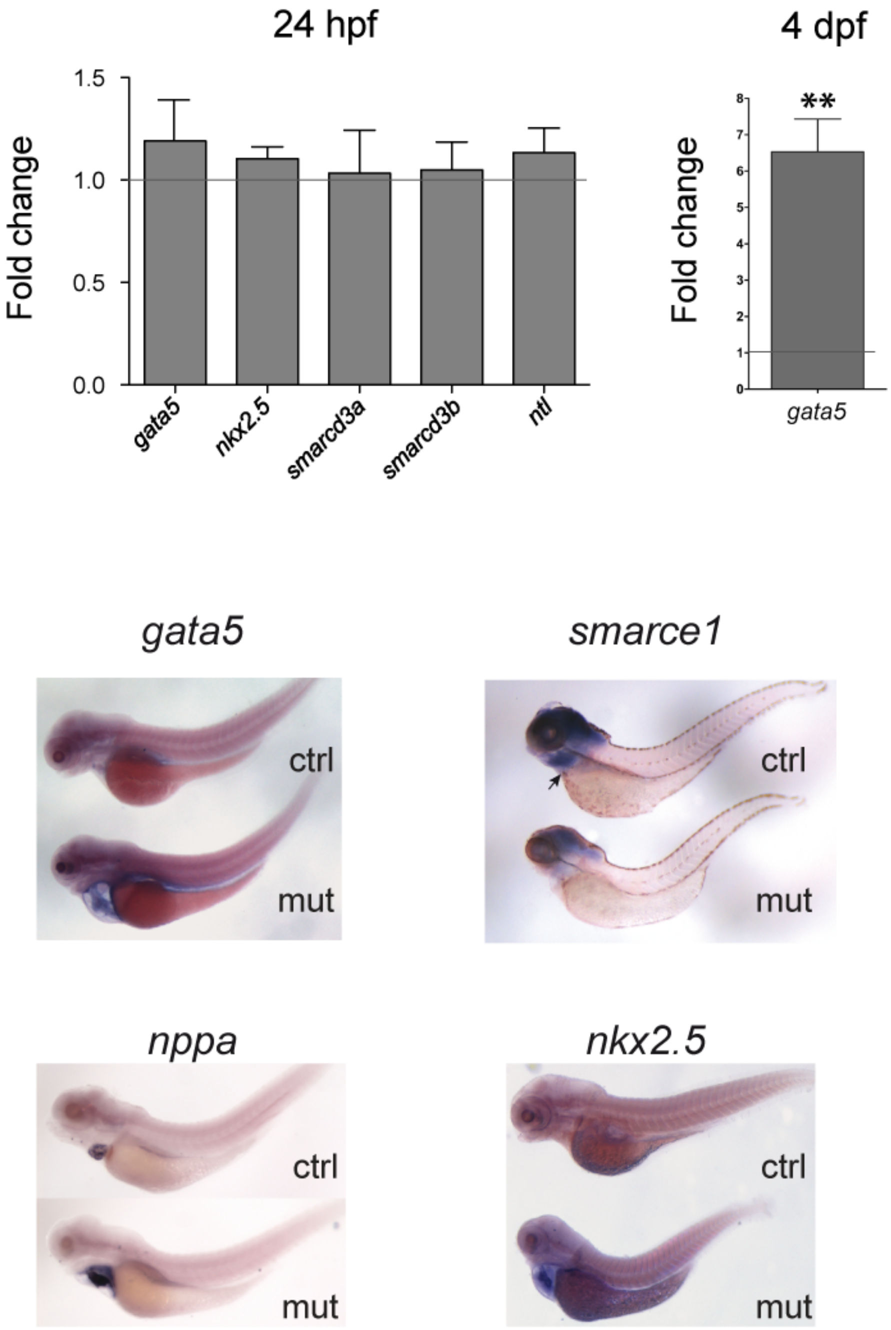


Supplementary figure 4.

Upper panel, left. Quantitation by qPCR of gene expression of *gata5, nkx2.5*, the *smarcd3* genes and *ntl*. 24 hpf embryos were genotyped and evaluated. For all genes n ≥3. No significant fold change was detected. Right. Quantitation by qPCR of *gata5* gene expression in isolated hearts of 4 dpf defective embryos compared to hearts of normal siblings indicated a significant difference (P value = 0,0087). Lower panel. Expression of *gata5, smarce1, nppa* and *nkx2.5* in 4 dpf (*smarce1*) and 5 dpf (all other markers) normal and defective larvae detected by ISH (lateral views).

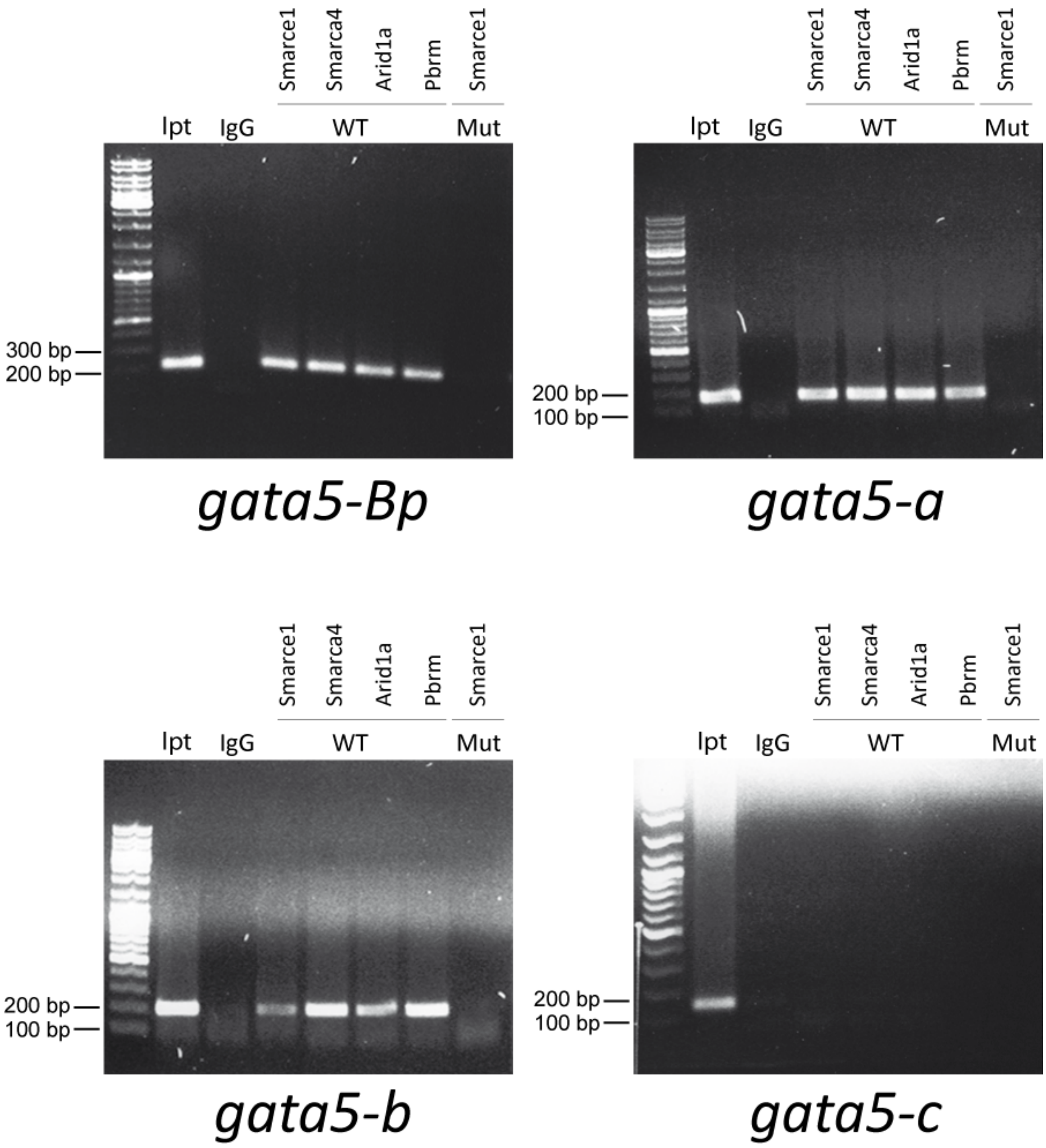


Supplementary figure 5.

Full-length gel images representing results from ChIP assays. Expected lengths for amplicons are: *gata5*-Bp, 217 bp; *gata5*-A,175 bp; *gata5*-B,197 bp; *gata5*-C,151 bp.

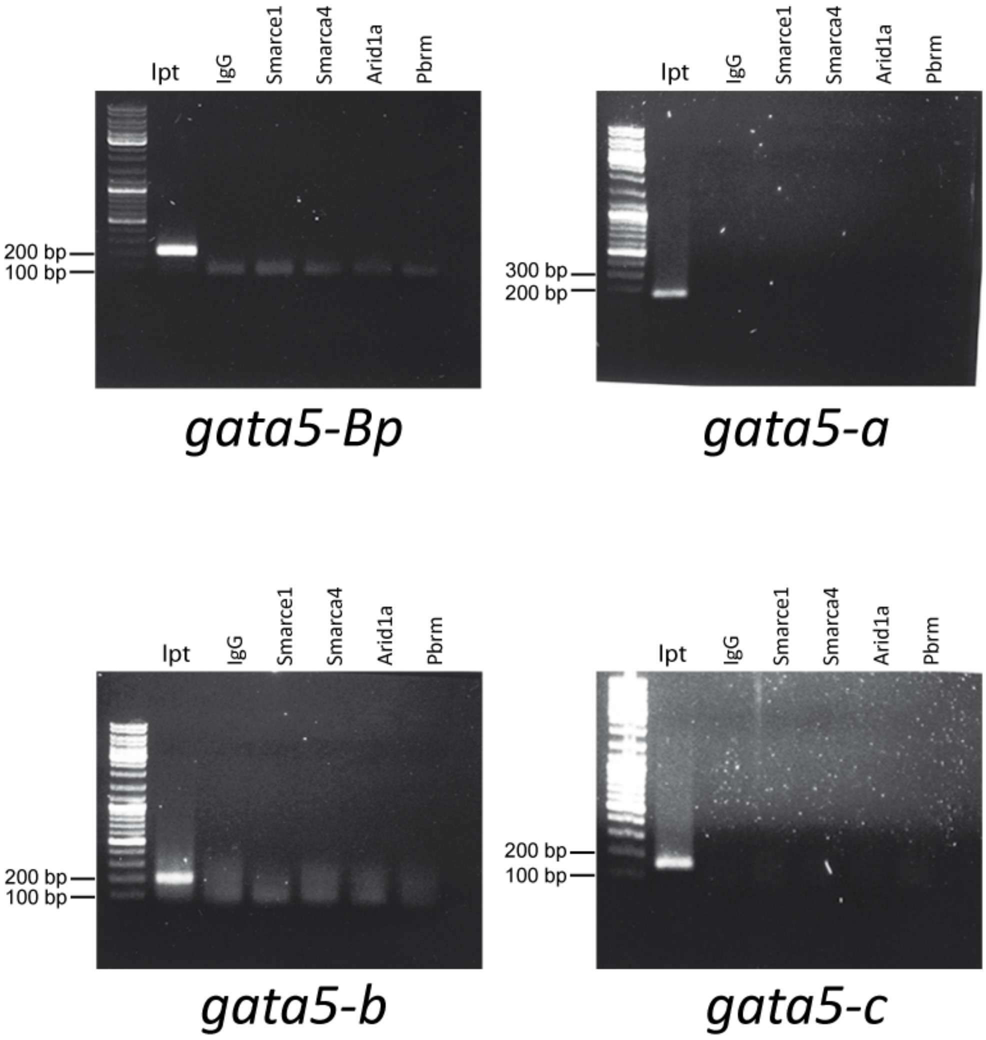


Supplementary figure 6.

Full-length gel images representing results from ChIP assays. Genomic DNA was obtained from mutant embryos. IgG, Smarce1, Smarca4, Arid1a and Pbrm indicate the PCR products from DNA precipitated using the corresponding antibody. Expected lengths for amplicons are: *gata5*-Bp, 217 bp; *gata5*-A,175 bp; *gata5*-B,197 bp; *gata5*-C,151 bp.
